# The effect of different levels of pneumoperitoneum pressures on regional cerebral oxygenation during robotic assisted laparoscopic prostatectomy

**DOI:** 10.3906/sag-2005-368

**Published:** 2021-06-28

**Authors:** Arzu KARAVELİ, Ali Sait KAVAKLI, Murat ÖZÇELİK, Mutlu ATEŞ, Kerem İNANOĞLU, Sadık ÖZMEN

**Affiliations:** 1 Department of Anesthesiology and Reanimation, Antalya Training and Research Hospital, University of Health Sciences, Antalya Turkey; 2 Department of Urology, Antalya Training and Research Hospital, University of Health Sciences, Antalya Turkey

**Keywords:** Near-infrared spectroscopy, pneumoperitoneum, prostatectomy, robotic **-** assisted surgery, trendelenburg position

## Abstract

**Background/aim:**

This study aimed to evaluate the effect of low- and high-pressure pneumoperitoneum pressures applied during robotic-assisted laparoscopic prostatectomy (RALP) using near-infrared spectroscopy (NIRS) on regional cerebral oxygenation saturation (rSO_2_).

**Materials and methods:**

The prospective, comparative, and observational study included patients aged 18–80 years, with the American Society of Anesthesiologists (ASA) physical status I-II, who would undergo elective RALP. The patients were divided into two groups (12 mmHg of pneumoperitoneum pressure group, n=22 and 15 mmHg of pneumoperitoneum pressure group, n=23). Patients’ demographic data, durations of anesthesia, surgery, pneumoperitoneum, and Trendelenburg position, intraoperative estimated blood loss, fluid therapy, urine output, hemodynamic and respiratory data, and rSO_2_ values were recorded at regular intervals.

**Results:**

The rSO_2_ values increased significantly during the pneumoperitoneum combined with steep Trendelenburg position (from *t* *3* to *t* *6* ) and at the end of the surgery ( *t* *7* ) in both groups, compared to the values 5 min after the onset of pneumoperitoneum in the supine position ( *t* *2* ) (P < 0.05), but no statistical significance was observed between the two groups. No cerebral desaturation was observed in any of our patients. Hemodynamic and respiratory parameters were preserved in both groups. The blood lactate levels were significantly higher in patients operated at high-pressure pneumoperitoneum, compared to those with low-pressure pneumoperitoneum (P < 0.05).

**Conclusion:**

We believe that low-pressure pneumoperitoneum, especially in robotic surgeries, such as robotic-assisted laparoscopic prostatectomy (RALP), can be applied safely.

## 1. Introduction

The steep Trendelenburg position is defined as the body tilted at an angle of 30 to 40°. It has been reported that the long-term pneumoperitoneum combined with steep Trendelenburg impairs cerebrovascular autoregulation, increases the risk of intracranial pressure (ICP) and cerebral edema, and may cause neurological deterioration secondary to hemodynamic changes such as increased mean arterial blood pressure and increased systemic vascular resistance [1]. There is, thus, an increasing interest in determining optimal pressure with minimal adverse effects of pneumoperitoneum since it provides adequate surgical workspace during laparoscopy [2]. In studies conducted to determine the optimal pneumoperitoneum pressure during RALP, it has been shown that, compared to the standard pneumoperitoneum pressure of 15 mmHg, 12 mmHg of pneumoperitoneum pressure does not cause postoperative complications [3] and reduces hospital stay and postoperative ileus rate [2]. 

It has been stated that during RALP, secondary to the pneumoperitoneum combined with steep Trendelenburg, cerebral blood volume and ICP increases, and consequently, cerebral edema, hypoperfusion, and ischemia may occur [4,5]. Therefore, additional methods are needed to enable early diagnosis and treatment of cerebral dysfunction during robotic surgery [5]. Jugular venous oxygen saturation (SjvO2) well illustrates the ratio of cerebral blood flow to cerebral metabolic rate, which is used as an indirect marker of cerebral metabolic oxygen rate. Yet, SjvO2 is invasive and difficult to use [6]. 

Near-infrared spectroscopy (NIRS) is a device that reflects the balance between cerebral oxygen consumption and demand and allows continuous and noninvasive monitoring of regional cerebral oxygenation saturation (rSO_2_) by using different absorption characteristics of oxygenated and deoxygenated hemoglobin (HbO2 and Hb, respectively) in cerebral oximeter [1,7]. Studies have shown that rSO_2_ values below 50% or less than 75% of the baseline values are associated with cerebral ischemia, postoperative cognitive dysfunction, and even longer hospital stay [8–10]. The NIRS has been utilized to observe whether or not the cerebral oxygen delivery is adequate in patients who undergo surgical procedures with a high risk of adverse neurological outcomes [10–12]. The NIRS has started to be applied for noninvasive evaluation of cerebral damage in many recent operations, where cerebral oxygenation is thought to be affected directly or indirectly, as in the case of surgical position [12–14]. It has been reported in the literature that although a decrease in rSO_2_ is associated with postoperative cognitive dysfunction [15], a reduction in rSO_2_ can improve with blood transfusion or 100% oxygen therapy [16]. However, steep Trendelenburg position and pneumoperitoneum administration must be avoided in patients with a history of cerebrovascular disorders or cerebral ischemia as the pneumoperitoneum pressure administered in the steep Trendelenburg position has also been reported to increase ICP exaggeratedly, and result in a disaster in such patients [17]. 

In the literature, few studies have investigated the intraoperative results of low- and high-pressure pneumoperitoneum during RALP. In this study, we aimed to evaluate the effects of different pneumoperitoneum pressures applied together with the steep Trendelenburg position on cerebral oxygenation by using NIRS in patients undergoing RALP due to prostate carcinoma.

## 2. Materials and methods 

### 2.1. Study design

This prospective, comparative, and observational study was approved by the ethics committee of the University of Health Sciences, Antalya Training and Research Hospital (No: 16/10). All patients were informed, and those patients whose written consents were obtained between January and July 2018 were included in the study. This study followed the Strengthening of the Reporting of Observational Studies in Epidemiology (STROBE) reporting guidelines [18].

Patients between the ages of 18 and 80, with a body mass index (BMI) of ≤40 kg/m2, with the American Society of Anesthesiology (ASA) score I-II, and those scheduled for elective RALP were included in the study. The patients were informed about the possible risks, benefits, and complications. The patients with the following conditions were excluded from the study: declined to give written informed consent, were under 18 or over 80 years of age, with an ASA score of III and above, with neurological disease and history of cerebrovascular disease, being treated for congestive heart failure, anemia, hematological disorder, and any metabolical disease, had acute intracranial vascular lesions (intracranial infection, head trauma, stroke, intracranial hemorrhage), requiring emergency surgery. The patients were divided into two groups: low-pressure pneumoperitoneum (Group I: 12 mmHg of pneumoperitoneum pressure; n = 22) and high-pressure pneumoperitoneum (Group II: 15 mmHg of pneumoperitoneum pressure; n = 23). 

### 2.2. Anesthesia protocol

As a routine, a preoperative evaluation was performed for RALP. The patients’ demographic data [age, body mass index (BMI), ASA physical status, comorbidity, and smoking status], durations of anesthesia, surgery, pneumoperitoneum, and Trendelenburg position, estimated amounts of intraoperative blood loss, intraoperative fluid therapy, and urine output, hemodynamic data [heart rate (HR), mean arterial pressure (MAP), peripheral oxygen saturation (SpO2)], rSO_2_ values (right and left), vasopressor support therapy and blood gas [hemoglobin (Hb), blood lactate levels, partial arterial oxygen (PaO2), and carbon dioxide (PaCO2)] values were recorded. Data collection was conducted by a blinded anesthesia nurse. The patient was carefully monitored during the operation, and the data including hemodynamic parameters, rSO_2_ values, blood gas values, fluid therapy, and urine output were recorded at regular intervals. While the data related to the study were recorded by a blinded anesthesia nurse in the anesthesia form, they were also recorded in a separate data collection form.

An 18 G vascular catheter was inserted in all patients taken to the operating room to start the IV fluid treatment. Right and left basal rSO_2_ values were recorded by placing a NIRS probe (Masimo Corp. Irvine, CA, USA) in the frontotemporal region of all patients. Following the premedication with 0.04 mg/kg IV midazolam, anesthesia was induced with 2 mcg/kg IV fentanyl, 2-3 mg/kg IV propofol, and 0.6 mg/kg IV rocuronium. Intraoperative ASA basic monitoring protocol was applied, including electrocardiogram (ECG), HR, noninvasive blood pressure (NIBP), SpO2, and end-tidal carbon dioxide (etCO2). The patients were intubated with the appropriate size of the endotracheal tube. Following the tracheal intubation, invasive arterial pressure (via 20 G artery catheter into a radial artery) (BD Arterial Cannula, Becton Dickinson, Utah, USA), urine output, and esophageal body temperature were monitored. The patients were ventilated in the volume-controlled mode. In order to reduce the negative effect of the pneumoperitoneum combined with the 30° steep Trendelenburg position on the lungs, ventilator settings were set with the tidal volume of 6–8 mL/kg and with the inspiratory/expiratory ratio of 1:2 and 4–7 cm H2O positive end-expiratory pressure (PEEP). To maintain an etCO2 within 30-35 mmHg, the tidal volume or the respiratory rate adjusted, appropriately (Primus, Drager, Luebeck, Germany). Anesthesia was maintained with 40% dry air-oxygen mixture and 0.8–1.5 age-adjusted minimum alveolar concentration (MAC) of desflurane. MAP was maintained within 20% of the pre-induction value in both groups by adjusting remifentanil infusion at 0.1–0.2 µg /kg/min IV. Muscle relaxation was achieved with 10 mg IV rocuronium at the discretion of the participating anesthesiologist and according to the surgeon’s needs. During anastomosis, fluid therapy was restricted (1 mL/kg/h) until ureterovesical anastomosis was completed in order to prevent blurring in the surgical site, and reduce the development of fascial, pharyngeal, and laryngeal edema in the steep Trendelenburg position [19]. After the ureterovesical anastomosis was completed and the patient was placed back in the supine position, a 1 L Ringer’s lactate bolus was administered, taking into account the patient’s volume. Twenty minutes before the surgery ended, all patients were administered 1000 mg IV paracetamol and 100 mg IV tramadol for postoperative analgesia in addition to ondansetron 4 mg IV infusion for antiemetic prophylaxis. At the end of the surgery, 0.05 mg/kg IV neostigmine and 0.02 mg/kg IV atropine were administered to antagonize the neuromuscular block. All patients were awakened in the operating room and then transferred to the recovery room. Postoperative analgesia was maintained with paracetamol for the first 72 h (1000 mg every 8 h, intravenously). The recovery time was defined as the time from the discontinuation of the inhalation agent and remifentanil at the end of the surgery to the removal of the tracheal tube [17].

The da-Vinci surgical robot (Intuitive Surgical, Mountain View, CA), designed to transform, filter, and transmit the surgeon’s hand motion, was used for RALP. All patients were placed in a modified lithotomy position during surgery after anesthesia induction. The arms were placed adjacent to the body. Shoulder pads were placed on the patients’ acromioclavicular joints. The pressure points were supported with soft pads. Pneumoperitoneum was obtained using an automatic insufflator (Olympus, Hamburg, Germany). During robotic and auxiliary port placement, the initial pneumoperitoneum was set to 15 mmHg. After the port was placed in the supine position, the patient was placed in the 30º Trendelenburg position. Pneumoperitoneum pressure was set to the desired level (15 mmHg or 12 mmHg) during the surgical procedure, at the surgeon’s own discretion [2].

Hypotension was defined as more than 20% decrease in basal MAP [20]. Hypotension was planned to be treated first with liquid boluses, followed by 5–10 mg IV ephedrine boluses. Vasopressor treatment was planned in patients when the treatment was insufficient. Perioperative Hb concentration was kept above 7 g/dL. Red blood cell transfusion was planned for patients with Hb concentration below 7 g/L. Hemodynamic parameters were recorded at baseline (5 min after induction) ( *t* *1* ), 5 min after the onset of pneumoperitoneum in the supine position ( *t* *2* ), during the combination of Trendelenburg position and pneumoperitoneum [at the 30th min ( *t3* ), the 60th min ( *t4* ), and the 120th min ( *t* *5* ) of pneumoperitoneum], and at the end of surgery ( *t* *6* ). Blood gas parameters were also recorded baseline ( *t* *1* ), 5 min after pneumoperitoneum in the supine position ( *t* *2* ), 30 min ( *t* *3* ), 60 min ( *t* *4* ), and 120 min ( *t* *5* ) after pneumoperitoneum in the Trendelenburg position, and at the end of the surgery ( *t* *6* ). 

### 2.3. Near-infrared spectroscopy (NIRS) measurement 

NIRS is a noninvasive imaging method that reflects the balance between supply and demand in cerebral oxygenation using different absorption characteristics of HbO2 and Hb [7]. NIRS is routinely performed in all robotic cases in our hospital to evaluate cerebral oxygenation. All patients were informed about the benefits and possible complications of NIRS in the preoperative period. NIRS sensors were placed in the frontotemporal area of all patients after the skin surface was cleaned with alcohol and before preoxygenation. The head was held in a neutral position with a silicone pillow to optimize the arterial and venous mixture. NIRS values were recorded before the anesthesia induction ( *t* *1* ), 5 min after the onset of pneumoperitoneum in the supine position ( *t* *2* ), during the combination of Trendelenburg position and pneumoperitoneum [at the 30th min ( *t3* ), the 60th min ( *t4* ), 90th min ( *t5* ), and at the 120th min ( *t* *6* ) of pneumoperitoneum], and at the end of surgery ( *t* *7* ). Cerebral desaturation was defined as a rSO_2_ value below 75% of the baseline value (80% if the baseline value was lower than 50%) for 15 s [17]. 

### 2.4. Primary and secondary aims

The primary aim of the study was to evaluate the effect of low- and high-pneumoperitoneum pressures applied during RALP on rSO_2_ using NIRS. The secondary aim was to observe hemodynamic and respiratory parameters.

### 2.5. Statistics analysis

Statistical analysis was performed using SPSS version 23 (SPSS Inc., Chicago, IL, USA). Categorical data are presented as absolute frequency ( *n* ) and percentage ( *%* ), while continuous data are presented as mean (standard deviation) or median (interquartile range). Normality analysis was performed using the Shapiro–Wilk test. Student t-test and Mann–Whitney U test were used for the analysis of independent groups. Categorical variables were analyzed using Pearson chi-square or Fisher’s exact test. Repeated measures of variance (ANOVA) was used to test any change in rSO_2_ among the study time points. Differences within the group were analysed with a paired t-test with Bonferroni correction. The P-value less than 0.05 was considered statistically significant.

The sample size was calculated using the G*power 3 analysis program (Heinrich-Heine Universitat Düsseldorf, Germany) before the study. A pilot study was carried out on 8 patients from each group. Power analysis was based on NIRS changes under 12 and 15 mmHg pressures of pneumoperitoneum. The mean rSO_2_ value was 56.90 (10.12) in the 12-mmHg pneumoperitoneum group, while it was 66.30 (6.01) in that of the 15 mmHg pneumoperitoneum group. The sample size was computed with a confidence interval of 95% and a significance level of 5%, and it was concluded that each group should consist of 19 patients to obtain statistically significant values. Considering the drop-out rate, it was estimated that there should be at least 22 patients in each group.

## 3. Results 

**Figure 1:**
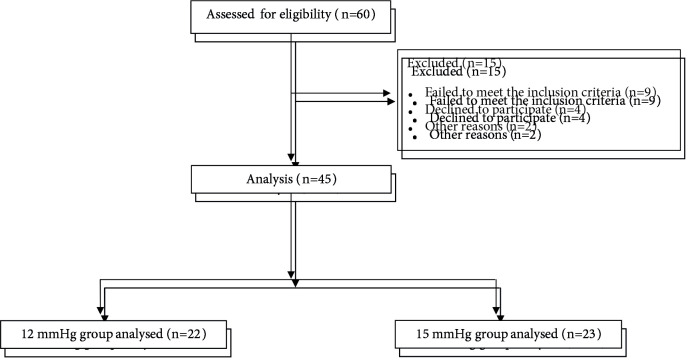
Patients’ flow chart.

A total of 60 patients were asked to participate in the study. Nine patients did not meet the inclusion criteria, four patients declined to participate in the study, and two surgeries were cancelled. Therefore, data from the remaining 45 patients, 22 in the 12-mmHg pneumoperitoneum group (Group I) and 23 in the 15-mmHg pneumoperitoneum group (Group II), were analyzed for the study (Figure 1).

Characteristics of the patients, including age, body mass index (BMI), ASA physical status, smoking, and comorbidities are summarised in Table 1.

**Table 1:** Demographic characteristics of patients.

	Group I (n = 22)	Group II (n = 23)
Age (y)	64.36 (4.86)	62.95 (5.65)
BMI (kg/m2)	27.74 (3.90)	28.69 (3.46)
ASA physical status, I/II	3/19	5/18
Smoking	3 (13.6%)	5 (21.7%)
Comorbidity	16 (72.7%)	13 (56.5%)

Data are presented as mean (standard deviation) or the number (percentage). BMI: body mass index, ASA: American Society of Anesthesiology.

No statistically significant differences were found between the two groups in terms of the duration of surgery and anesthesia (P = 0.339 and P = 0.399, respectively). Moreover, duration of pneumoperitoneum and Trendelenburg were similar (P = 0.419 and P = 0.356, respectively). No patient needed vasopressor therapy or a bolus of ephedrine in the intraoperative period in both groups. The intraoperative estimated blood loss was similar (P = 0.255). None of the patients required blood transfusion. No statistically significant difference was found between the two groups in terms of intraoperative fluid therapy (P = 0.126) and urine output (P = 0.729) (Table 2).

**Table 2:** Intraoperative data of patients.

	Group I (n = 22)	Group II (n = 23)	P-value
Fluid therapy (mL)	1500(1300–2000)	1600(1500–2000)	0.126
Urine output (mL)	350(275–450)	400(300–400)	0.729
Estimated blood loss (mL)	100(100–150)	100(80–150)	0.255
Time (min)			
Anesthesia	252(228–300)	240(210–270)	0.399
Surgery	222(198–267)	205(180–240)	0.339
Pneumoperitoneum	200(170–230)	175(155–215)	0.419
Trendelenburg	180(150–212.5)	160(130–200)	0.356

Data are presented as median (interquartile range); *P <0.05 is statistically significant.

No statistically differences were found between the two groups in terms of changes in rSO_2_. However, when the rSO_2_values were compared with the values 5 min after the onset of pneumoperitoneum in the supine position ( *t* *2* ); it was observed that the right and the left rSO_2_ values increased significantly during the pneumoperitoneum combined with steep Trendelenburg position (from *t* *3* to *t* *6* ) and at the end of the surgery ( *t* *7* ) in both groups (Table 3). No patient had a rSO_2_ value <75% of the baseline value for ≥15 s.

**Table 3:** Regional cerebral oxygenation saturation changes of patients.

Left rSO_2_				Right rSO_2_		
	Group I (n = 22)	Group II (n = 23)	P-value	Group I (n = 22)	Group II (n = 23)	P-value
t1	65.54 (9.60)	65.69 (9.61)	0.958	66.86 (9.64)	64.69 (8.88)	0.437
t2	63.13 (10.17) a,b,c,d,e	64.95 (7.13) f,g,h,i,j	0.794	62.50 (10.08)k,l,m,n,o	62.91 (8.22) p,r,s,t,u	0.982
t3	68.13 (12.26) a*	70.91 (9.57) f*	0.633	68.54 (9.10) k*	71.00 (12.19) p*	0.450
t4	69.18 (12.35) b*	73.43 (8.89) g*	0.191	68.95 (8.45) l*	70.69 (11.31) r*	0.563
t5	69.27 (10.59) c*	73.78 (9.219 h*	0.134	69.36 (7.43) m*	71.17 (11.22) s*	0.529
t6	70.95 (9.76) d*	73.78 (8.53) i*	0.306	70.68 (6.40) n*	71.60 (10.91) t*	0.731
t7	70.86 (9.55) e*	73.60 (11.059 j*	0.379	70.81 (7.62) o*	71.17 (9.75) u*	0.893

Data are presented as mean (standard deviation); *P <0.05 is statistically significant. rSO_2_: regional cerebral oxygenation saturation. a P = 0.005 t2 vs t3, b P = 0.001 t2 vs t4, c P = 0.000 t2 vs t5, d P = 0,001 t2 vs t6, e P = 0.000 t2 vs t7, f P = 0.000 t2 vs t3, g P = 0.001 t2 vs t4, h P = 0.002 t2 vs t5, i P = 0.002 t2 vs t6 ,j P = 0.000 t2 vs t7, kP = 0.000 t2 vs t3, l P = 0.000 t2 vs t4, m P = 0.000 t2 vs t5, n P = 0.000 t2 vs t6, o P = 0.002 t2 vs t7, p P = 0.000 t2 vs t3, r P = 0.001 t2 vs t4, s P = 0.001 t2 vs t5, t P = 0.000 t2 vs t6, u P = 0.000 t2 vs t

Hemodynamic changes of patients are shown in Table 4. In terms of HR and MAPs, no statistical significance was observed in all time periods, and both HR and MAP were maintained during RALP in both groups. Although the SPO2 value was higher in Group I, compared to Group II, no statistically significant difference was found between the two groups. 

**Table 4:** Hemodynamic findings of patients.

	Group I (n = 22)	Group II (n = 23)	P-value
Heart rate (beat/min)			
t1	76.68 (12.19)	73.39 (11.87)	0.364
t2	66.59 (17.38)	64.69 (15.21)	0.776
t3	62.68 (15.42)	59.82 (10.44)	0.811
t4	59.54 (12.90)	58.21 (9.28)	0.820
t5	61.54 (12.20)	60.08 (10.84)	0.991
t6	62.77 (11.59)	62.08 (13.31)	0.829
t7	63.90 (13.61)	61.52 (11.38)	0.526
Mean arterial pressure (mmHg)			
t1	88.40 (19.37)	85.08 (14.99)	0.522
t2	89.77 (21.05)	97.47 (17.84)	0.192
t3	90.00 (11.33)	92.60 (10.18)	0.421
t4	81.86 (9.78)	88.47 (12.13)	0.051
t5	81.95 (8.10)	86.47 (10.79)	0.120
t6	82.77 (8.50)	88.95 (11.79)	0.051
t7	70.40 (14.11)	77.78 (12.45)	0.070
Pulse oximetry (%)			
t1	98.90 (1.34)	98.78 (1.27)	0.615
t2	98.95 (1.21)	98.78 (0.99)	0.384
t3	98.68 (1.49)	98.13 (1.79)	0.295
t4	98.63 (1.43)	98.17 (1.69)	0.348
t5	98.68 (1.28)	98.43 (1.30)	0.444
t6	98.63 (1.04)	98.52 (1.37)	0.906
t7	98.90 (1.01)	98.60 (1.23)	0.463

Data are presented as mean (standard deviation); *P <0.05 is statistically significant.

**Figure 2:**
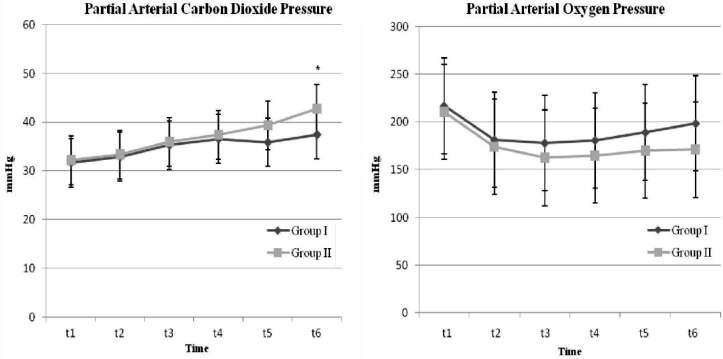
Partial arterial carbon dioxide and oxygen pressures changes of patients. Data are presented as mean (standard deviation); *P < 0.05 is statistically significant.

Although the blood lactate levels were at normal levels in both groups, when compared between the groups, it was observed that the blood lactate levels were statistically significantly higher in patients operated at high-pressure pneumoperitoneum ( *t* *2* , *t* *3* , *t* *4* , and *t* *5* ; P values were 0.028, 0.032, 0.041, and 0.027, respectively) (Table 5). Although Hb levels of patients were lower in Group I compared to Group II, they were not statistically significant (Table 6). In both groups, PaCO2 values started to increase from the onset of pneumoperitoneum, and peak at the end of the surgery ( *t* *6* ; P = 0.012). Although PaO2 values were lower in Group II, they were maintained in both groups during the study (Figure 2). 

**Table 5:** Lactate levels of patients.

	Group I (n = 22)	Group II (n = 23)	P-value
t1	1.2 (0.9–1.5)	1.2 (1–1.7)	0.793
t2	1.1 (1–1.4)	1.6 (1.1–1.8)	0.028*
t3	1.0 (0.8–1.4)	1.5 (1–1.8)	0.032*
t4	1.0 (0.8–1.4)	1.4 (0.9–1.8)	0.041*
t5	1.1 (0.8–1.4)	1.5 (0.9–1.5)	0.027*
t6	1.3 (1–1.6)	1.6 (1.1–2.0)	0.351

Data are presented as median (interquartile range); *p<0.05 is statistically significant.

**Table 6:** Hemoglobin levels of patients.

	Group I (n = 22)	Group II (n = 23)	P-value
t1	13.41 (1.17)	13.28 (1.30)	0.782
t2	12.44 (1.53)	13.17 (1.01)	0.064
t3	12.64 (1.64)	13.16 (0.85)	0.510
t4	12.63 (1.59)	12.98 (0.99)	0.380
t5	12.63 (1.66)	12.70 (1.12)	0.881
t6	12.63 (2.18)	12.94 (1.27)	0.564

Data are presented as mean (standard deviation); *P < 0.05 is statistically significant.

## 4. Discussion

Our study showed that rSO_2_ values increased significantly compared to the values obtained 5 min after the onset of pneumoperitoneum in the supine position, during the pneumoperitoneum combined with steep Trendelenburg position (from *t* *3* to *t* *6* ), and at the end of the surgery (t7) in both groups. There was no statistical significance between the rSO_2_ values in both groups. No cerebral desaturation was observed in any of our patients. Throughout the surgery, the blood lactate levels were significantly higher in patients who underwent high-pressure pneumoperitoneum than those who had low-pressure pneumoperitoneum. On the other hand, despite the Hb levels being lower in patients undergoing low-pressure pneumoperitoneum, there was no significant difference observed between the groups. 

In our study, although no significant difference was detected for the effect of different pneumoperitoneum pressures applied during RALP on rSO_2_ levels, the rSO_2_ values were observed to increase from the onset of pneumoperitoneum in patients with both low- and high-pressure pneumoperitoneum. This increase in rSO_2_ levels can be explained by an increase in cerebral blood flow and secondary to an increase in PaCO2 levels. Although our study found out that the hemodynamic parameters of the patients were preserved, it was also stated in the literature that the increase in MAP may contribute to the increase in cerebral blood flow during Trendelenburg [17]. In many studies, it has been reported that, when applied alone or in combination, Trendelenburg position and pneumoperitoneum increased ICP, which is related to increased venous pressure, cerebral blood volume, and cerebrospinal fluid volume that obstruct cerebral venous drainage [21,22]. Besides, it has been shown that pneumoperitoneum pressure applied during laparoscopic surgery prevents venous return from lumbar venous plexuses by increasing catecholamine release, independent of PaCO2, increasing ICP, and cerebral blood flow [17]. Cerebral blood flow can range from 2.7 to 8.0 kPa in line with the change in PaCO2 [23]. Park et al. evaluated the effect of pneumoperitoneum on cerebral oxygenation in the steep Trendelenburg position with NIRS in patients undergoing RALP and showed that rSO_2_ values increased significantly from the 30th min. of pneumoperitoneum to the end of surgery [17], which is consistent with our results. Another study also reported that rSO_2_ values increased significantly, from 70% to 73% during RALP [24]. A number of studies conducted with a focus on the correlations between rSO_2_ and ICP revealed that the rSO_2_ decreased as cerebral perfusion pressure declined due to increased ICP [11, 25]. 

Although hemodynamic changes caused by carbon dioxide insufflation are well tolerated, they are frequently seen in patients undergoing laparoscopic surgery. The cardiovascular complication rate in patients who undergo laparoscopic surgery may increase as a result of creating a pneumoperitoneum. Yet, it has been shown that lower intra-abdominal pressure levels are feasible and safe, besides reducing the cardiovascular consequences of a high-pressure carbon dioxide pneumoperitoneum [26]. A study by Ekici et al., which evaluated the cardiac effects of different intraabdominal pressures in laparoscopic cholecystectomy, showed that MAP and HR increased significantly in patients who were applied both low- and high-pressure pneumoperitoneum, compared to the baseline values, though there was no significant difference between the two groups [26]. As a matter of fact, our study found out that hemodynamic parameters were preserved in patients who were applied both low- and high-pressure pneumoperitoneum.

Cerebral oxygen saturation shows the balance between cerebral oxygen consumption and demand and is affected by heat, cerebral blood flow, Hb level, and cerebral metabolic rate [7]. Cerebral oximetry provides information about the blood flow below its location, under the localization of the probe [7]. In our study, the NIRS probe was placed in the frontotemporal region of the patients and thus, information was obtained only about the regional oxygenation of the frontotemporal cortex. Therefore, the increase in cerebral oxygen saturation in this study should not imply an increase in normal global oxygenation [7]. Cerebral desaturation did not occur in any patients in our study, which is an especially important finding for patients who will undergo major surgery at an advanced age. It has been reported in the literature that the incidence of cerebral desaturation is 26% in elderly patients who underwent major abdominal surgery [15].

Many factors may affect the surgical site during laparoscopic surgery [27]. It is generally thought that higher pressure pneumoperitoneum can provide a better view of the surgical site [28,29]. However, high-pressure pneumoperitoneum has not only hemodynamic but also undesirable surgical effects on the patient [30]. It has been reported in the literature that low-pressure pneumoperitoneum can be applied reliably [31], and because of the higher abdominal wall compliance in the low-pressure pneumoperitoneum [32], it can provide a better surgical view compared to high-pressure pneumoperitoneum [27]. In like manner, it was observed in our study that low-pressure pneumoperitoneum not only preserved the hemodynamics of the patients but also provided surgeons with sufficient vision. The European Association for Endoscopic Surgery recommends the use of the lowest intraabdominal pressures to achieve the surgical view for the sake of patient safety, rather than the use of routine intraabdominal pressures in laparoscopic surgeries [33].

However, it is also believed that using lower pressure pneumoperitoneum during laparoscopic surgery may decrease surgical visualization and complicate surgical dissection, thereby increasing the risk of the duration of surgery, blood loss, or organ damage [3, 31]. Yet, studies have indicated that there is no significant difference in low- and high-pressure pneumoperitoneum in terms of duration of surgery, hospital stay, and intraoperative blood loss [3]. No significant difference was found in our study between the durations of surgery, anesthesia, pneumoperitoneum, and Trendelenburg position, or estimated blood loss in patients undergoing low- and high-pressure pneumoperitoneum. Although Hb levels were lower in patients with low-pressure pneumoperitoneum, no statistically significant difference was found between the two groups, and no intraoperative blood transfusion was required in any of our patients. Despite the prolonged duration of surgery in patients undergoing low-pressure pneumoperitoneum, it is not statistically significant. 

High intraabdominal pressures may cause lactic acid accumulation in patients undergoing prolonged laparoscopic procedures. Taura et al. compared the different levels of intraabdominal pressures (15 vs. 10 mmHg) undergoing laparoscopic sigmoidectomy and found that blood lactate levels were higher in patients with high-pressure pneumoperitoneum [34]. Although blood lactate levels were within normal limits in both groups in our study, blood lactate levels were found to be higher in patients who were applied high-pressure pneumoperitoneum. Increase in blood lactate levels may result from increased anaerobic metabolism due to tissue ischemia caused by high-pressure pneumoperitoneum [35].

This study has several limitations. First, the study was planned prospectively but observationally. Prospective and randomized controlled studies are needed. Second, our study was planned on male patients, and could not be generalized to the general population or geriatric population. Thirdly, since the study was planned observationally, no tests were performed to evaluate the cognitive dysfunctions of the patients in the postoperative period.

In conclusion, although it was found that rSO_2_ values increased in patients undergoing RALP, there was no significant difference between the low- and high-pressure pneumoperitoneum. It was found that hemodynamic and respiratory parameters were better maintained in patients undergoing low-pressure pneumoperitoneum. Contrary to what is stated, low-pressure pneumoperitoneum did not significantly increase bleeding rates. Also, despite low- and high-pressure pneumoperitoneum having a similar effect on hemodynamic parameters, it was observed that low-pressure pneumoperitoneum has a more positive effect on blood lactate levels. For these reasons, we believe that low-pressure pneumoperitoneum can be applied reliably, especially in robotic surgeries such as RALP. However, further studies with a larger sample size are required to confirm the results of this study.

## Informed consent

Approval was obtained from the Ethics Committee of the University of Health Sciences, Antalya Training and Research Hospital (No: 16/10). Written informed consents were obtained from the patients.
